# Impact of Azithromycin on the Quorum Sensing-Controlled Proteome of *Pseudomonas aeruginosa*

**DOI:** 10.1371/journal.pone.0147698

**Published:** 2016-01-25

**Authors:** J. E. Swatton, P. W. Davenport, E. A. Maunders, J. L. Griffin, K. S. Lilley, M. Welch

**Affiliations:** 1 Department of Biochemistry, Tennis Court Road, Cambridge, CB2 1QW, United Kingdom; 2 Cambridge Centre for Proteomics, Tennis Court Road, Cambridge, CB2 1QW, United Kingdom; 3 Cancer Research UK Cambridge Institute, Li Ka Shing Centre, Robinson Way, Cambridge, CB2 0RE, United Kingdom; 4 Department of Pathology, Tennis Court Road, Cambridge, CB2 1QP, United Kingdom; 5 MRC Human Nutrition Research, Elsie Widdowson Laboratory, 120 Fulbourn Road, Cambridge, CB1 9NL, United Kingdom; Ghent University, BELGIUM

## Abstract

The macrolide antibiotic, azithromycin (AZM), has been reported to improve the clinical outcome of cystic fibrosis patients, many of whom are chronically-infected with *Pseudomonas aeruginosa*. However, the highest clinically-achievable concentrations of this drug are well-below the minimum inhibitory concentration for *P*. *aeruginosa*, raising the question of why AZM exhibits therapeutic activity. One possibility that has been raised by earlier studies is that AZM inhibits quorum sensing (QS) by *P*. *aeruginosa*. To explicitly test this hypothesis the changes brought about by AZM treatment need to be compared with those associated with specific QS mutants grown alongside in the same growth medium, but this has not been done. In this work, we used quantitative 2D-difference gel electrophoresis and ^1^H-NMR spectroscopy footprint analysis to examine whether a range of clinically-relevant AZM concentrations elicited proteomic and metabolomic changes in wild-type cultures that were similar to those seen in cultures of defined QS mutants. Consistent with earlier reports, over half of the AZM-induced spot changes on the 2D gels were found to affect QS-regulated proteins. However, AZM modulated very few protein spots overall (compared with QS) and collectively, these modulated proteins comprised only a small fraction (12–13%) of the global QS regulon. We conclude that AZM perturbs a sub-regulon of the QS system but does not block QS *per se*. Reinforcing this notion, we further show that AZM is capable of attenuating virulence factor production in another Gram-negative species that secretes copious quantities of exoenzymes (*Serratia marcescens*), even in the absence of a functional QS system.

## Introduction

*Pseudomonas aeruginosa* is Gram-negative pathogen that displays virulence towards a range of hosts including insects, nematodes, plants and some mammals [1–4]. The organism is responsible for up to 10% of all hospital-acquired infections, and is of particular concern for immunocompromised individuals, where it can cause pneumonia and serious systemic infections [5]. However, *P*. *aeruginosa* is probably best known for its role in cystic fibrosis (CF), where it is a leading cause of morbidity and mortality [6,7].

Since its introduction in 1991, there has been increased interest in use of the semi-synthetic azalide, azithromycin (AZM) for the treatment of chronic respiratory infections. Several clinical studies have shown that long-term treatment with AZM significantly improves lung function and body weight in CF patients [8,9]. AZM is thought to act by binding to the 23S rRNA in the 50S ribosomal subunit, thereby blocking the peptide exit channel. Consistent with this, mutations in the 23S rRNA-encoding gene lead to AZM resistance [10]. However, it is unlikely that this explains all of the therapeutic effect(s) of AZM because the minimal inhibitory concentration (MIC) of this drug for clinical *P*. *aeruginosa* isolates is typically around an order of magnitude higher (128 to 512 μg/ml [11] than the maximum clinically-achievable sputum concentrations (0.6 to 79.3 μg/ml [12]). Moreover, several studies have suggested that low (sub-MIC) concentrations of AZM appear to have a secondary effect; they suppress motility, biofilm formation and virulence factor production by *P*. *aeruginosa* [13–17]. Given that in *P*. *aeruginosa*, the production of many secreted virulence factors is under the control of quorum sensing (QS), one economical hypothesis has been that sub-MIC AZM acts by inhibiting this signalling mechanism [18–20]. Consistent with this notion, sub-MIC AZM reduces the production of *N*-acylhomoserine lactone (AHL) QS signal molecules and also decreases transcription of the synthases (*lasI* and *rhlI*) that make these molecules [18,19]. Interestingly though, Skindersoe *et al* have also shown that structurally-unrelated antibiotics (including the β-lactam, ceftazidime and the fluoroquinolone, ciprofloxacin) also strongly impinge upon QS [21] suggesting that QS might be exquisitely-sensitive to certain types of antibiotic-induced stress.

The QS system in laboratory-grown cultures of *P*. *aeruginosa* is hierarchical, being comprised of a two-tiered cascade with the RhlI-RhlR component subordinate to the LasI-LasR component. LasI is an *N*-3-oxododecanoyl-L-homoserine lactone (OdDHL) synthase. OdDHL is a small molecule which binds to a transcriptional regulator called LasR, thereby increasing the ability of the latter to promote (or more rarely, repress) the transcription of genes preceded by DNA sequences called *las* boxes [22,23]. *Las* boxes display only poor sequence conservation [24] but are associated with many exoproduct-encoding genes and secretory operons, so las signalling gives rise to increased secretion of tissue-degrading exoenzymes. LasR-OdDHL also further stimulates the expression *lasI*, giving rise to a positive induction loop. Moreover, the LasR-OdDHL complex also stimulates the transcription of two downstream regulators, *rhlR* and *pqsR*. RhlR is a transcription factor that binds to “*rhl* boxes” located upstream of another set of exoproduct-encoding genes, and like LasR, it also stimulates the expression of its cognate AHL synthase, *rhlI*. RhlI is an *N*-butanoyl-L-homoserine lactone (BHL) synthase. BHL binds to RhlR, further stimulating its ability to bind to DNA. LasR and RhlR also regulate a third arm of the QS system. The *Pseudomonas* quinolone signal (PQS) links the las and rhl signalling pathways (reviewed in [25,26]). LasR-OdDHL stimulates *pqsR* expression, whereas RhlR-BHL suppresses this. It is not yet clear how the PQS signalling pathway leads to increased virulence factor production [27,28]. Mutants defective in QS are less virulent *in vivo* and secrete fewer virulence factors than their wild-type counterpart [29]. More recently, Pérez-Martinez and Haas have suggested that sub-MIC AZM may impinge on QS by reducing the expression of a key regulator of *P*. *aeruginosa* QS: *gacA* [30]. GacA is an activator of *lasR* and *rhlR* expression, and *gacA* mutants show impaired BHL production as well as reduced expression of the global RNA regulators, *rsmZ* and *rsmY* [31–33]. Confusingly though, sub-MIC AZM was also found to inhibit the expression of *rsmA*, whose protein product (RsmA) antagonizes rsmZ/rsmY action, so it is not clear how AZM affects the truly critical cellular parameter of ([rsmZ] + [rsmY])/[RsmA].

Interestingly, the suppression of virulence factor production caused by sub-MIC AZM is still dependent upon AZM binding to the ribosome. Heterologous expression of 23S rRNA methylase (ErmBP from *Clostridium perfringens*, or ErmC from *Staphylococcus aureus*) in *P*. *aeruginosa* leads to methylation of a crucial adenine residue, thereby preventing AZM from accessing the peptide exit channel. This methylation not only confers resistance to the bactericidal action of AZM (i.e., increases the MIC); it also restores virulence factor [34] and biofilm [35] production in the presence of sub-MIC AZM. Furthermore, biochemical pull-downs using immobilized AZM were dominated by ribosome-associated proteins [35]. Collectively, these data strongly suggest that the principle (if not only) target of AZM in the cell is the ribosome. However, the premature peptide chain termination brought about by AZM also has more subtle effects—by blocking elongation, AZM leads to the release of covalently-bonded peptidyl-tRNA complexes (a phenomenon known as “drop off”). As these complexes accumulate, they deplete the pool of free tRNA [8,36]. Commensurate with this, over-expression of the endogenous *P*. *aeruginosa* peptidyl-tRNA hydrolase, Pth, was found to counteract many of the inhibitory effects associated with AZM [37]. Perhaps not unexpectedly, “drop off” has a disproportionate effect on proteins enriched in rare codons, especially if these encode the first 2–6 amino acids in the protein. Interestingly, codon 2 in *rhlR* is a rarely-used arginine codon (AGG), and replacement of this with a more common Arg codon (CGC) partially reversed the inhibition of rhamnolipid production brought about by sub-MIC AZM treatment [37]. This observation immediately provides a possible rationale as to how sub-MIC AZM might affect a specific subset of mRNA molecules.

In 2006, Nalca *et al* investigated the impact of sub-MIC AZM on the transcriptional and proteomic profile of *P*. *aeruginosa* [20]. They did this by examining the changes brought about by exposure of *P*. *aeruginosa* cultures to a single sub-MIC concentration (2 μg/ml) of AZM. In doing this, they identified around 107 transcripts and 43 proteins whose expression was modulated by AZM. By comparing their lists of AZM-modulated transcripts and proteins with previously-published lists of QS-regulated transcripts proteins, these workers concluded that sub-MIC AZM exhibits extensive antagonism of QS. However, and on a note of caution, the same authors also recognised the considerable inter-study variability of the QS regulon, and commented on the difficulties of comparing functional genomic datasets generated by different groups using different growth conditions. For example, Nalca *et al* used brain-heart infusion broth as a growth medium in their AZM work, whereas their comparator datasets were derived from cultures grown in different media: Nouwens *et al* investigated the effect of QS on the proteome of cultures grown in LB medium [38] and Arevalo-Ferro *et al* investigated the QS-regulated proteome in ABt minimal medium supplemented with sodium citrate and casamino acids [39]. Given that the QS regulon is known to be affected by the growth medium employed [40], this complicates any cross-study comparisons that can be made.

In this work, we set out to explicitly test the hypothesis that AZM inhibits QS in *P*. *aeruginosa*, and to establish which of the las, rhl or PQS branches of the signalling system was most affected. In particular, we examined whether treatment of wild-type *P*. *aeruginosa* cultures with a range of clinically-relevant AZM concentrations caused proteomic and metabolomic changes similar to those observed in isogenic QS mutants grown alongside in the same growth medium in the absence of AZM. Our results show that AZM influences the expression of some, but not all QS-regulated proteins, and that these effects are accompanied by a significant number of QS-independent effects. We also show that the presence of a QS system is not necessary for AZM to suppress virulence. We conclude that AZM should not be considered to be a generic QS inhibitor, although it does affect a specific sub-regulon of the QS system.

## Materials and Methods

### Bacterial strains and growth conditions

Wild-type PAO1, PDO100 (a *rhlI*::Tn501-2 derivative of PAO1 [41]) and PAO-JP1 (a *lasI*::Tet derivative of PAO1 [42]) were generously provided by Barbara Iglewski (University of Rochester, New York). MP551 (a *pqsR*::IS*phoA*/hah-Tc derivative of PAO1 [43]) was a gift from Colin Manoil (University of Washington Center for Genome Sciences, Seattle, USA). PA0230 (*lasB*::*lacZ*) and PA0217 (*rhlA*::*lacZ*) are mini-CTX*lacZ* derivatives described in [44]. The mini-CTX*lacZ* (*cdrA*) construct was made by PCR-amplifying the *cdrA*-*hprA* intergenic region (upstream of *cdrA*) using primers 5’-GATCAAGCTTCTATCTGCGTGGCGCACGTCAG-3’ and 5’-GATCGGATCCGGAAGGTTCCTTGGCGGCAGCG-3’. The resulting ca. 320 bp amplicon (which encompasses the transcriptional start site of *cdrA* [45]) was digested with HindIII and BamHI and ligated to similarly-digested mini-CTX*lacZ*. The ligation mixture was introduced into *E*. *coli* strain JM109 by electroporation. Tet^R^ transformants were selected on 10 μg/ml tetracycline and confirmed by sequencing across the insert. The resulting mini-CTXlacZ (*cdrA*) was introduced into PAO1 by electroporation. Tet^R^ transformants were selected on 50 μg/ml tetracycline and confirmed as previously described [44]. The *Serratia marcescens* strains Sma12 and Sma274 were generously supplied by George Salmond (University of Cambridge) [46].

*P*. *aeruginosa* was grown in alanine-glycerol-salts (AGS) medium [47] (56 mM alanine, 17 mM K_2_HPO_4_, 85 mM NaCl, 0.1 mM CaCl_2_, 10 mM MgSO_4_, 5 μM FeCl_2_, 7.5 μM ZnCl_2_ and 0.5% v/v glycerol, pH 7.0) in the presence or absence of AZM (2, 8 or 32 μg/ml concentration, as indicated, and added at the start to each culture). This medium is compatible with both proteomic and metabolomic analyses. Liquid cultures were inoculated at an initial OD_600_ of 0.001 and grown with vigorous aeration at 37°C in baffled flasks. Azithromycin was a generous gift from Pfizer Limited, Kent (UK).

### Protease assay

*P*. *aeruginosa* strain PAO1 was grown in AGS medium in the presence or absence of AZM (32 μg/ml). Culture supernatant samples were collected at the indicated time points throughout the growth curve and assayed for protease activity using azocasein as a substrate, as described previously [48]. Protease activity was expressed as ΔA_436_/h/ml. Secreted protease was measured in the same way for cultures of *Serratia marcescens* except that the cultures were grown in LB ± 10 μg/ml AZM.

### Beta-galactosidase assay

The *lasB*::*lacZ* (PA0230) and *rhlA*::*lacZ* (PA0217) reporter strains were grown at 37°C in AGS medium in the presence or absence of AZM (32 μg/ml) as indicated. The β-galactosidase activity associated with cell pellets harvested throughout the growth curve was assayed using the chromogenic substrate, *O*-nitrophenyl-β-D-galactopyranoside, as previously described [48]. Enzyme activity was expressed as the initial rate per millilitre of sample per OD_600_ (ΔA_420_/min/ml/OD_600_).

### Preparation of samples for 2-Dimensional Fluorescence Difference in-Gel Electrophoresis (2D-DiGE)

#### (i) Extracellular fraction

Two and half hours after entry into stationary phase, bacterial cells were separated from the culture supernatant by sedimentation (8,000 × *g*, 20 min, 4°C). Note that although the growth curves of the different QS mutants were similar in overall profile (i.e., they all exited exponential phase growth at around the same time), they each reached different final optical density values. At the time of harvesting, the PAO1 and AZM-treated cultures reached an OD_600_ of 6.5, whereas the *lasI*, *rhlI* and *pqsR* mutants reached final OD_600_ values of 2.7, 4.9 and 3.5, respectively. The cell pellets from 500 ml culture were immediately frozen and stored at -80°C. Culture supernatants (400 ml volume) were passed through a 0.2 μm filter (Millipore Stericup) and trichloroacetic acid (TCA) was added to a final concentration of 10% w/v. After 4 hr at 4°C, the precipitated protein was collected by sedimentation (8,000 × *g*, 30 min, 4°C) and the pellet was washed three times with 80% acetone to remove residual TCA. Each pellet was resuspended in ASB14 lysis buffer (8 M urea, 2% w/v amido-sulfobetaine 14, 5 mM magnesium acetate, 20 mM Tris-HCl, pH 8.5) supplemented with protease inhibitor cocktail set I (Calbiochem, La Jolla, CA, USA). The pH of the protein samples was re-adjusted to pH 8.5 using NaOH and the protein concentration was determined using the Bio-Rad DC protein assay kit.

#### (ii) Cell-associated fraction

The frozen cell pellets were resuspended on ice in TE lysis buffer (50 mM Tris-HCl, 4 mM EDTA, pH 8.3) supplemented with a protease inhibitor cocktail (Roche) and phenylmethylsulfonyl fluoride (0.1 mM). The cell suspension was sonicated (3 × 10 sec bursts, MSE microtip, maximum power output) and centrifuged (15,000 × *g*, 30 min, 4°C) to remove particulate debris and unlysed cells. Proteins were then phenol extracted as previously described [47].

### 2-Dimensional Fluorescence Difference in-Gel Electrophoresis (2D DiGE)

2D DiGE was performed essentially as previously described [47]. Briefly, four independent biological replicates of the cell-associated and extracellular protein samples from each of the 7 strains/conditions examined (i.e., untreated wild-type PAO1, *lasI* mutant (PAO-JP1), *rhlI* mutant (PDO100), *pqsR* mutant (MP551), and wild-type cultures treated with 2, 8 and 32 μg/mL AZM) were labelled with CyDye DIGE Fluor Minimal Dyes (GE Healthcare). To avoid potential dye-related artefacts, for each condition, two samples were labelled with Cy3 and two with Cy5. A pooled sample, containing equal amounts of all 28 cell-associated or extracellular samples was labelled with Cy2 and included in all of the corresponding gels. This ensured that all protein spots were represented in all of the gels. We used pH 4–7 zoom strips and 24 cm SDS-PAGE gels to resolve the cell-associated proteome, and pH 3–10 non-linear (NL) strips with 13 cm SDS-PAGE gels for the extracellular spots. Following electrophoresis, gels were scanned for Cy3, Cy5 and Cy2 fluorescence using a Typhoon^™^ 9400 device (GE Healthcare). Protein expression was quantified using DeCyder Biological Variation Analysis V5.02 (BVA, GE Healthcare). Protein spots showing significant changes (p≤0.01) in PAO1 treated with the different concentrations of AZM and/or in the QS mutants (with respect to the untreated wild-type) were picked from a colloidal Coomassie-stained gel and digested with trypsin (MassPrep Station, Micromass). The resulting peptides were analysed using LC-MS/MS (QTof2, Micromass) and the data output was used to identify proteins from primary sequence databases (NCBI) using the Mascot search engine (www.matrixscience.com/).

### Univariate and multivariate analysis of proteomics data

Following gel-to-gel matching of protein spots, statistical analysis (Student’s t-test) of normalized protein abundance changes between sample groups was carried out using DeCyder BVA, as previously described [47]. In addition, the data were also subjected to multivariate analyses using SIMCA-P 10 (Umetrics, Umea, Sweden). Only protein spots that were reliably detected in at least 75% of the gels and which had a spot volume (pixel intensity) of >10^5^ were included in the analysis. The filtered *cell-associated* protein dataset contained 776 spots with 28 observations for each, while the corresponding *extracellular* dataset contained 621 spots with 28 observations for each. Principal Components Analysis (PCA) was used to cluster the data from the cell-associated and extracellular 2-D DiGE studies. The corresponding loadings plots were used to identify those protein spots that contributed most towards the observed separation between the QS mutants, the AZM-treated samples and the untreated controls. The most discriminatory protein spots giving rise to separation in the scores plots were removed iteratively in groups of 10 and the models were re-built on the reduced datasets [49]. Proteins ranked as being within the top 60 most discriminatory spots and only contributing to separation of AZM-treated PAO1 from the untreated control (in addition to those identified by univariate analysis) were subsequently picked from a colloidal Coomassie-stained gel for identification purposes.

### High resolution ^1^H-NMR spectroscopy metabolomic analysis

Culture supernatants (5 mL volume) harvested 2.5 hours after the culture had entered stationary phase were filtered on ice (Sartorius Minisart Sterile EO filters, 0.2 μm pore size) and frozen at -80°C. These supernatant samples and standard solutions of 128 μg/mL AZM in H_2_O (1 mL each) were lyophilized and then reconstituted in 500 μL D_2_O (Goss Scientific Instruments) containing 5 mM sodium (3-trimethylsilyl)-2,2,3,3-tetradeuteriopropionate (TSP) (Cambridge Isotope Laboratories, Inc.). The samples were analyzed using a 500 MHz Bruker Avance spectrometer with a triple axis inverse (TXI) proton carbon probe. Spectra were acquired and processed as previously described [50], using a solvent suppression pulse sequence based on the start of the Nuclear Overhauser Effect SpectroscopY (NOESY) pulse sequence termed a NOESY presat pulse sequence (relaxation delay = 2 s; spectral width = 5707 Hz; time domain = 32,768 data points). The following spectral regions were excluded from data analysis: (i) region 4.56–5.24 ppm, encompassing the water peak; (ii) all regions found to be above baseline in the 128 μg/mL AZM standards spectra (3.97–4.02, 3.89–3.95, 3.57–3.65, 3.42–3.56, 3.31–3.38, 3.17–3.28, 2.80–2.96, 1.45–1.50, 1.18–1.41, 1.02–1.08 and 0.86–0.93 ppm). This was done to prevent resonances corresponding to AZM contributing to the PCA models.

## Results

### The effects of sub-MIC AZM on total protease activity and transcription of QS-regulated genes

**Fig 1:**
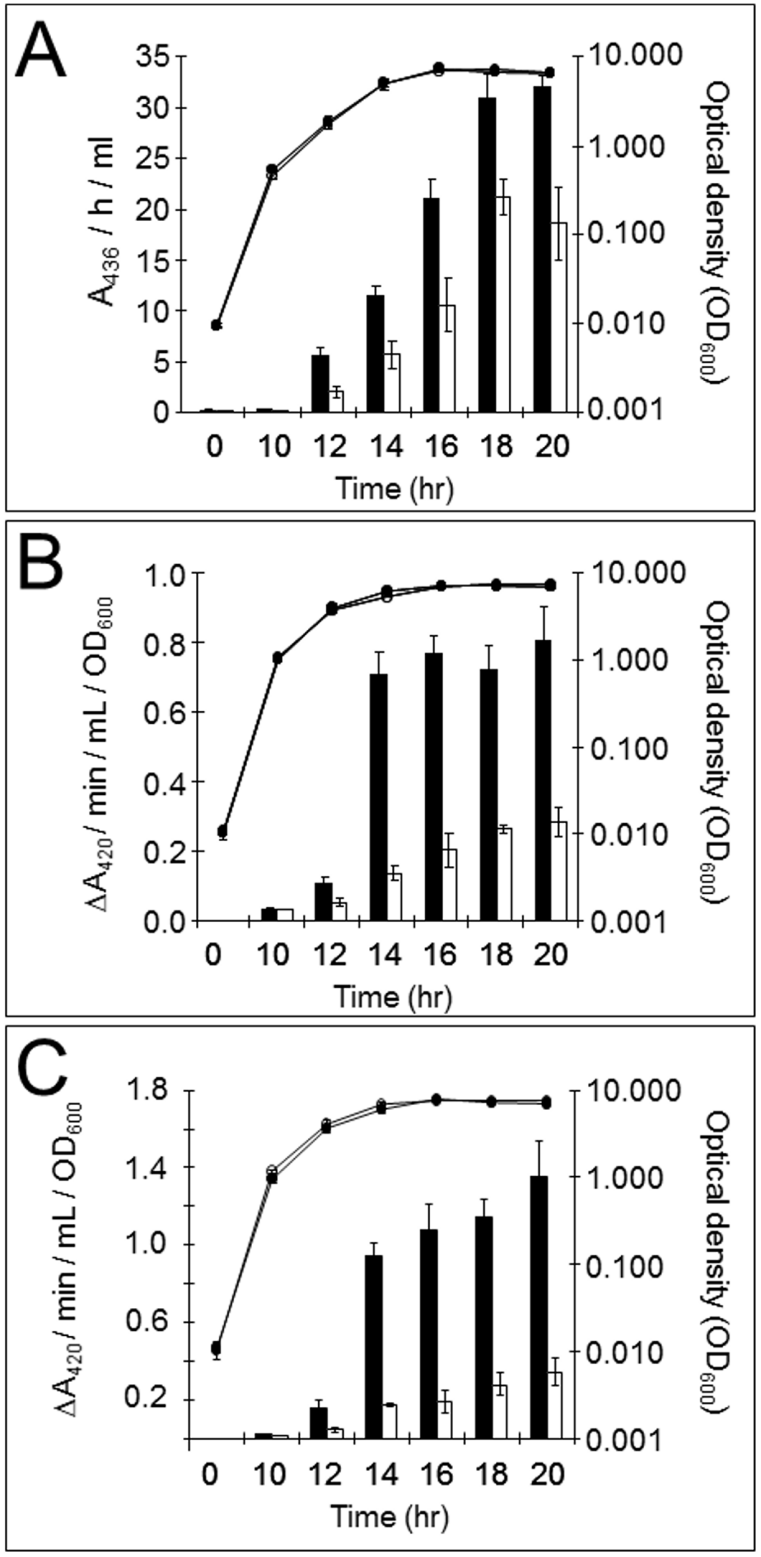
Phenotypes affected by AZM in *P*. *aeruginosa*. **(A)** AZM reduces secreted protease production. PAO1 cultures were grown in the absence (filled bars/symbols) or in the presence (open bars/symbols) of AZM (32 μg/ml). Samples were harvested throughout the growth curve (circles) and supernatants were assayed for protease activity (bars). Results are means ± SD of 3 independent experiments. **(B)** Effect of AZM on the transcription of *lasB*. Strain PA0230, which contains a chromosomal *lasB*:*lacZ* transcriptional fusion, was grown in the absence (closed bars/symbols) or in the presence (open bars/symbols) of AZM (32 μg/ml). Samples were harvested throughout the growth curve (circles) and assayed for β-galactosidase activity (bars). Results are means ± SD of 3 independent experiments. **(C)** Effect of AZM on the transcription of *rhlA*. As in (B), except that β-galactosidase activity was measured in PA0217, which carries a chromosomal *rhlA*:*lacZ* transcriptional fusion.

As a first step, and to confirm that AZM elicits similar effects in our defined growth medium (AGS) compared with earlier studies, we examined the effects of sub-inhibitory concentrations of AZM on secreted protease activity and on the transcription of two genes (*lasB* and *rhlA*) known to be under QS control. The MIC of AZM for PAO1 grown in AGS was measured using E-test strips and found to be >256 μg/ml. No discernible effects on growth rate were apparent at concentrations ≤32 μg/ml. AZM (32 μg/ml) decreased the total secreted protease activity of a PAO1 culture and depressed the β-galactosidase activity derived from *lasB*:*lacZ* and *rhlA*:*lacZ* transcriptional fusions without any discernible effect on the growth rate of the cultures (Fig 1). We conclude that AZM affects QS-regulated phenotypes in AGS medium at clinically-achievable concentrations of the drug.

### Analysis of cell-associated and extracellular protein expression in PAO1 (± AZM)

**Fig 2:**
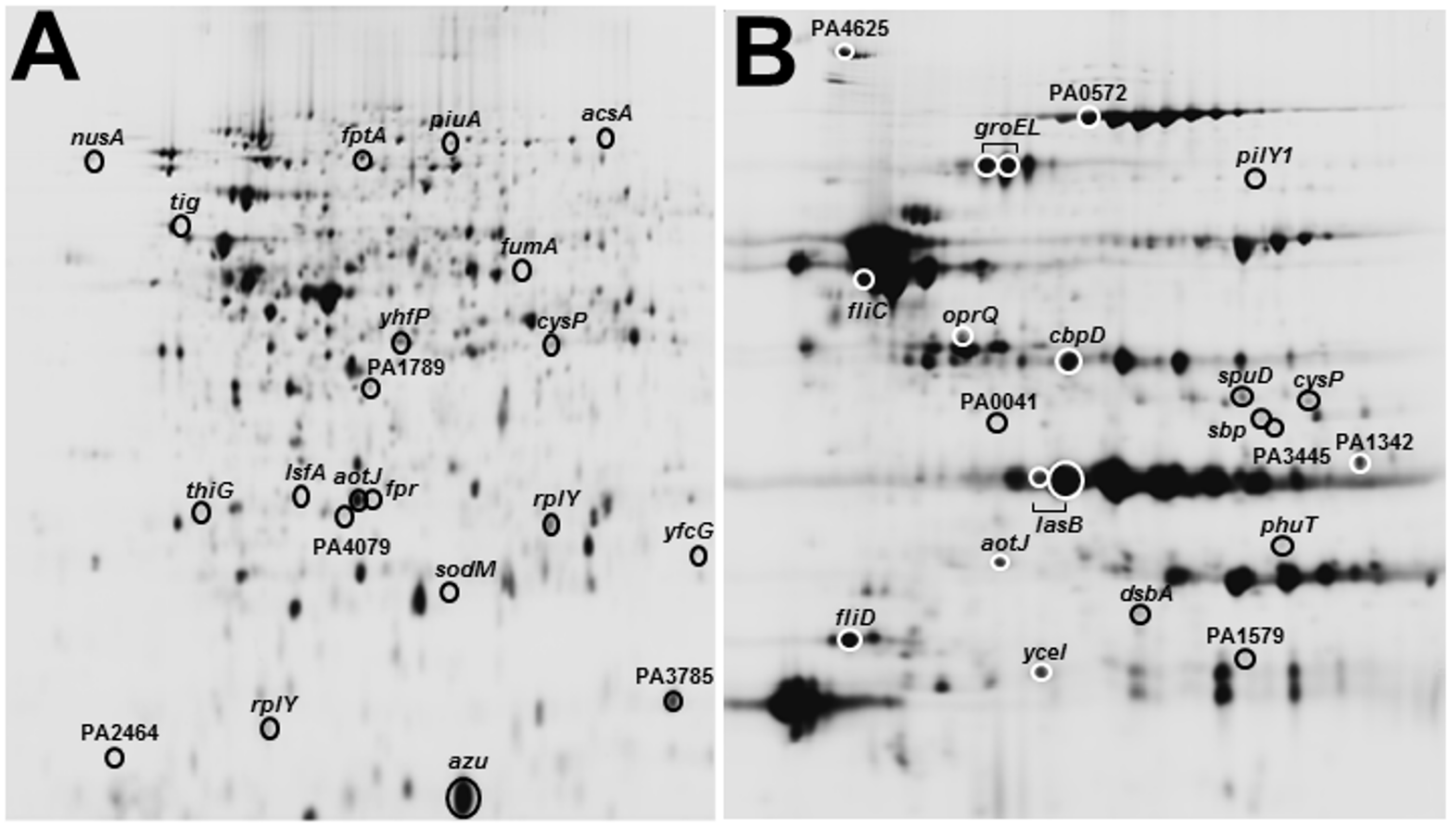
Typical intracellular and extracellular protein profiles of *P*. *aeruginosa*. Extracted proteins (here, from a pooled sample of proteins from the wild-type, *lasI*, *rhlI* and *pqsR* mutant cultures), were labelled with CyDye and separated by 2-D gel electrophoresis on 24 cm pH 4–7 linear gradient strips for the intracellular proteins (panel **A**) or 13 cm pH 3–10 non-linear gradient strips for the extracellular proteins (panel **B**) followed by electrophoresis on 12% SDS-polyacrylamide gels. Proteins were visualised by scanning for CyDye fluorescence at the appropriate wavelengths. The position of the identified protein spots that were modulated by AZM are indicated (see Results and Discussion).

We used 2D DiGE coupled with DeCyder Biological Variation Analysis (BVA, GE Healthcare) to quantify protein expression in PAO1 ± sub-MIC AZM (2, 8 or 32 μg/ml). For each concentration of AZM examined, four independent biological replicates were analysed. The protein profiles for the cell-associated and secretome fractions are shown in Fig 2. Treatment of PAO1 with AZM yielded discrete spot changes in both the cell-associated and extracellular fractions, compared with the untreated control (summarised in Table 1). In the cell-associated proteome, most modulations were 2-fold or less. The effects of AZM on the extracellular proteome were more pronounced, with most modulated proteins being altered in abundance by >2-fold. The polyamine transport protein, SpuD, and the chaperone, GroEL, was modulated to the greatest degree by AZM, being 4.8-fold (p = 0.0065) and 8.7-fold (p = 0.06; spot identified through PCA) increased in expression, respectively. Although the data in Table 1 indicate that 8 μg/ml AZM has a more pronounced effect on the proteome than either 2 or 32 μg/ml AZM, this is a consequence of the conventional (but arbitrary) threshold (p≤0.01) applied in the univariate t-test analysis; multivariate analyses (see below) indicated that AZM elicited a concentration-dependent continuum of proteomic effects, most of which fell below the p-value threshold considered significant in the univariate analyses. This observation reinforces the importance of using multiple statistical approaches for data analysis.

**Table 1:** Effect of QS and AZM on the proteome of *P*. *aeruginosa*.

	Total number of intracellular spot changes	↓	↑	Total number of extracellular spot changes	↓	↑
2 μg/ml AZM	2	2	0	2	1	1
8 μg/ml AZM	22	16	6	20	13	7
32 μg/ml AZM	17	16	1	11	3	8
*lasI*	100	39	61	115	60	55
*rhlI*	30	10	20	60	47	13
*pqsR*	38	29	9	45	24	21

The table shows the number of proteins that were significantly modulated (p≤0.01) in each of the different QS mutants (the *lasI* mutant (PAO-JP1), the *rhlI* mutant (PDO100) and the *pqsR* mutant (MP551)) and in wild-type strain (PAO1) treated with the indicated sub-MIC AZM, compared with untreated PAO1.

### Effects of QS on cell-associated and extracellular protein profiles

**Fig 3:**
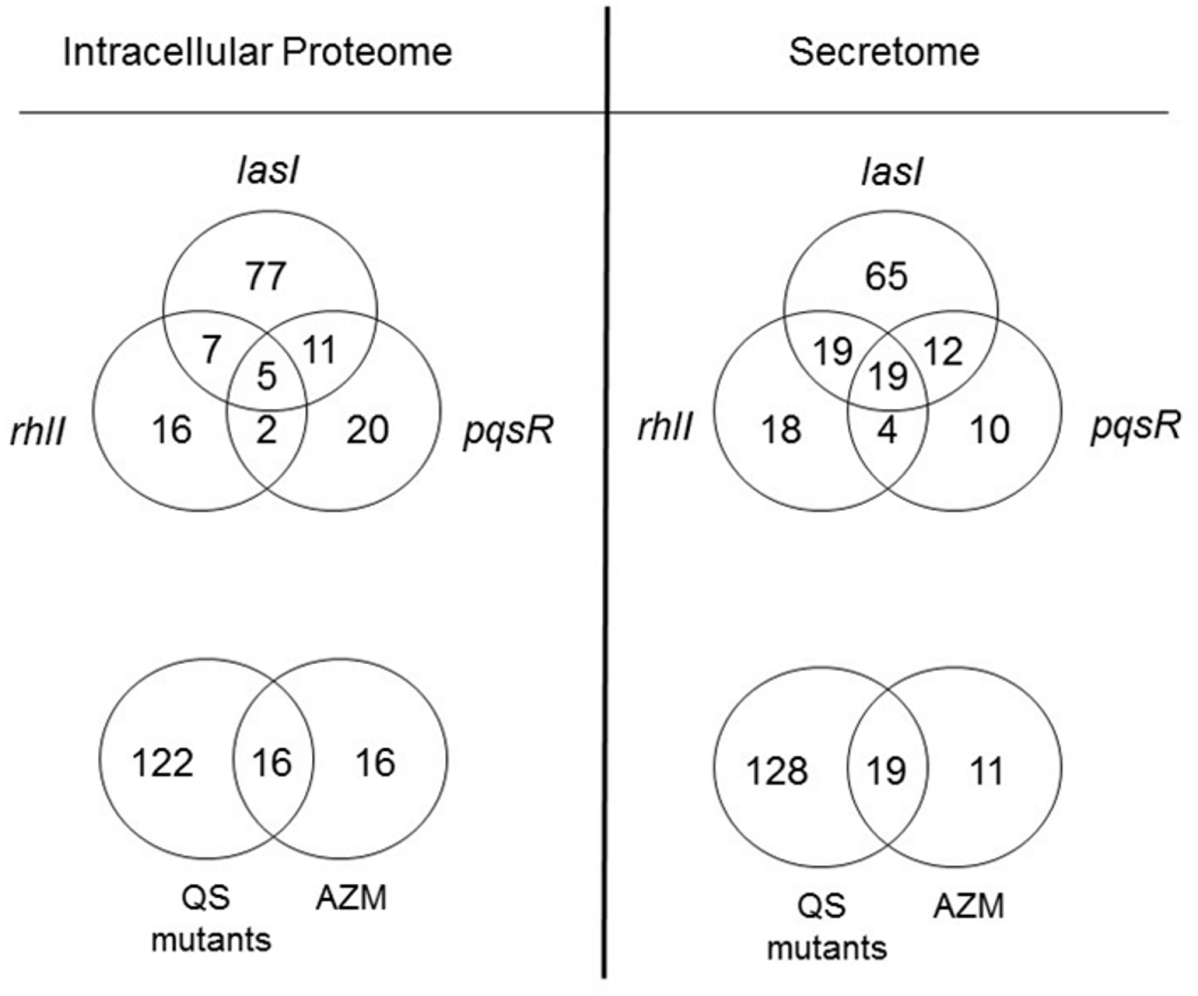
Overlap(s) between the QS and AZM-modulated proteome. The figure shows Venn diagrams indicating inter-relationships between the proteins that were significantly (p≤0.01) modulated in the QS mutants (*lasI*, *rhlI* and *pqsR* mutants, as indicated) compared with wild-type PAO1 (upper panels) or between the spots modulated collectively in the QS mutants and PAO1 samples treated with all concentrations of AZM (lower panels).

In parallel with the AZM treatment, we also characterized the proteome in a *lasI* mutant (PAO-JP1), a *rhlI* mutant (PDO100) and a *pqsR* mutant (MP551). Mutants in *lasI* are unable to make OdDHL, whereas mutants in *rhlI* and *pqsR* are unable to make BHL and PQS, respectively. The total number of spot changes (p≤0.01) observed for each of the QS mutants compared with wild-type PAO1 is shown in Table 1. In both the cell-associated and extracellular proteome, the largest number of modulated proteins occurred in the *lasI* mutant, which is consistent with this component of the signalling system being located at the top of the signalling hierarchy. In the extracellular proteome, the majority of spots showed decreased expression for all three QS mutants (Table 1). Fig 3 (upper panels) shows the overlap of modulated proteins observed for each of the QS mutants. Only a relatively small number of protein spots were modulated by all three QS sub-systems, and many proteins were uniquely altered in abundance in each of the mutants.

### Effects of AZM on QS

Although sub-MIC AZM altered the abundance of relatively few proteins (32 spots were modulated in the cell-associated proteome and 30 spots in the secretome) over half of these spots were also modulated in the QS mutants (Fig 3, lower panels). Overall though, AZM had an impact on only a small fraction of the QS-modulated proteome; just 12% of the QS-regulated cell-associated proteome was affected by AZM, and 13% of the QS-regulated secretome. There was no correlation between the volume of the QS-regulated spots and their modulation by AZM, indicating that our analysis was not influenced by the overall abundance of any given QS-regulated protein. We conclude that AZM has a significant impact on a small portion of the QS-controlled proteome of *P*. *aeruginosa*. Further inspection of the data revealed that AZM primarily affected secreted proteins controlled by the rhl signalling system (15 out of the 19 AZM-modulated spots (p≤0.01) in the secretome that overlapped with the QS regulon were also affected in the *rhlI* mutant (S1 Fig)). However, it is also worth noting that sub-MIC AZM modulated non-QS-controlled proteins (Fig 3, lower panels).

### Multivariate analysis of proteomic data

**Fig 4:**
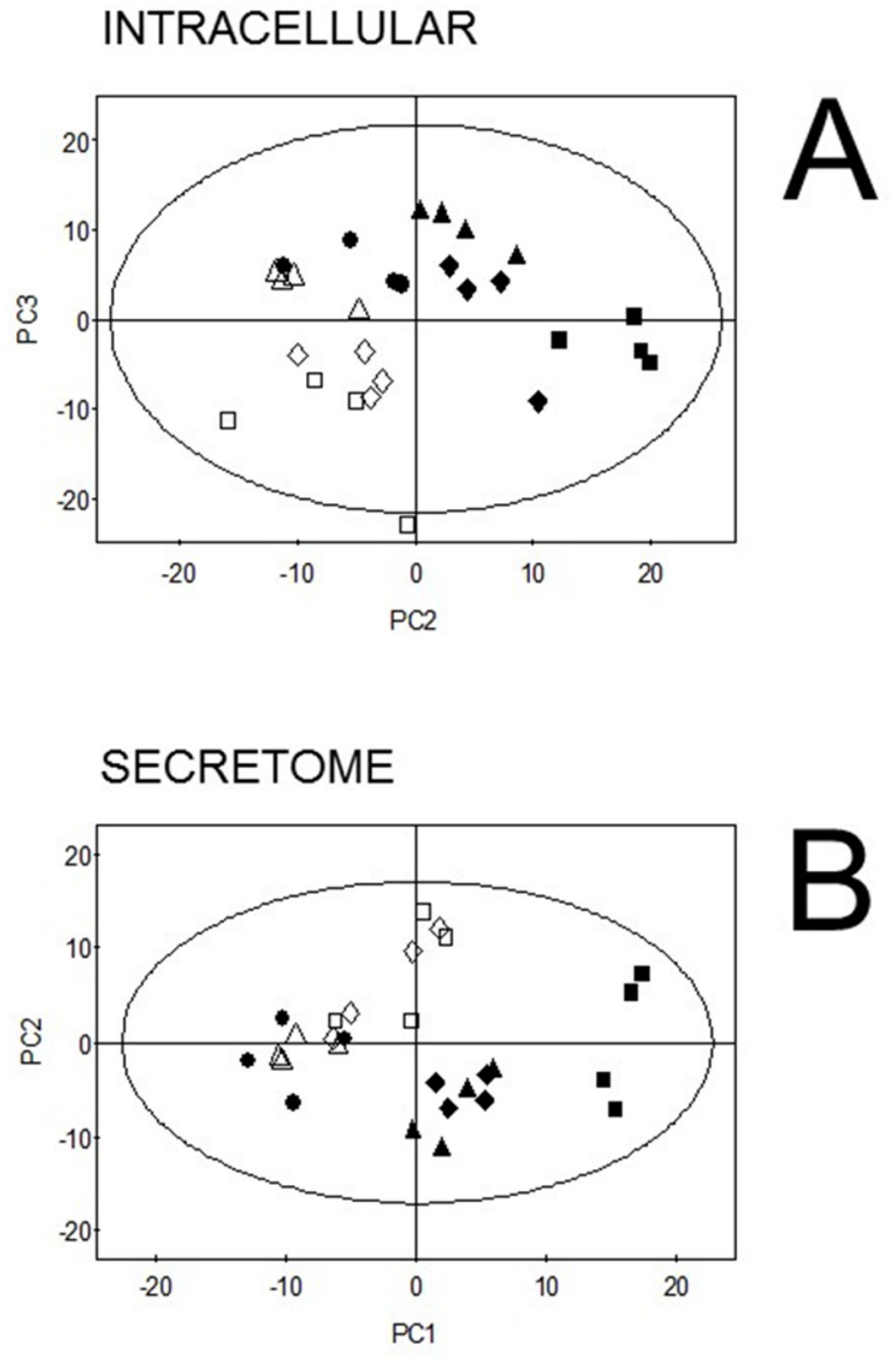
PCA scores plots of proteomics data from the intracellular and extracellular fractions. Data generated in DeCyder BVA from the intracellular (panel **A**) and secreted (panel **B**) proteome were subjected to Principle Components Analysis (PCA), as described in *Materials and Methods*. **Symbols**: *lasI* mutant ■, *rhlI* mutant ▲, *pqsR* mutant ◆, untreated PAO1 ●, PAO1 treated with 2 μg/ml AZM △, PAO1 treated with 8 μg/ml AZM ◊, PAO1 treated with 32 μg/ml AZM □. Note that 4 biological replicates are represented for each sample type.

It is possible that we under-estimated the impact of sub-MIC AZM on the QS regulon due to an over-reliance on univariate t-testing as a means of assessing the statistical significance of spot modulations. Therefore, and to identify subtle or potentially latent trends in the data, we used PCA. Neither the cell-associated nor extracellular proteome PCA scores plot showed pronounced co-clustering of the AZM-treated samples with those of the different QS mutants (Fig 4). Furthermore, removal of the 60 most discriminatory spots from the PCA models did not substantially affect the clustering of either the QS mutants or the AZM-treated samples (*data not shown*), indicating that multiple protein spots contribute incrementally towards the observed separations. These observations support the notion that sub-MIC AZM does not globally affect QS.

### ^1^H-NMR metabolomic analysis

**Fig 5:**
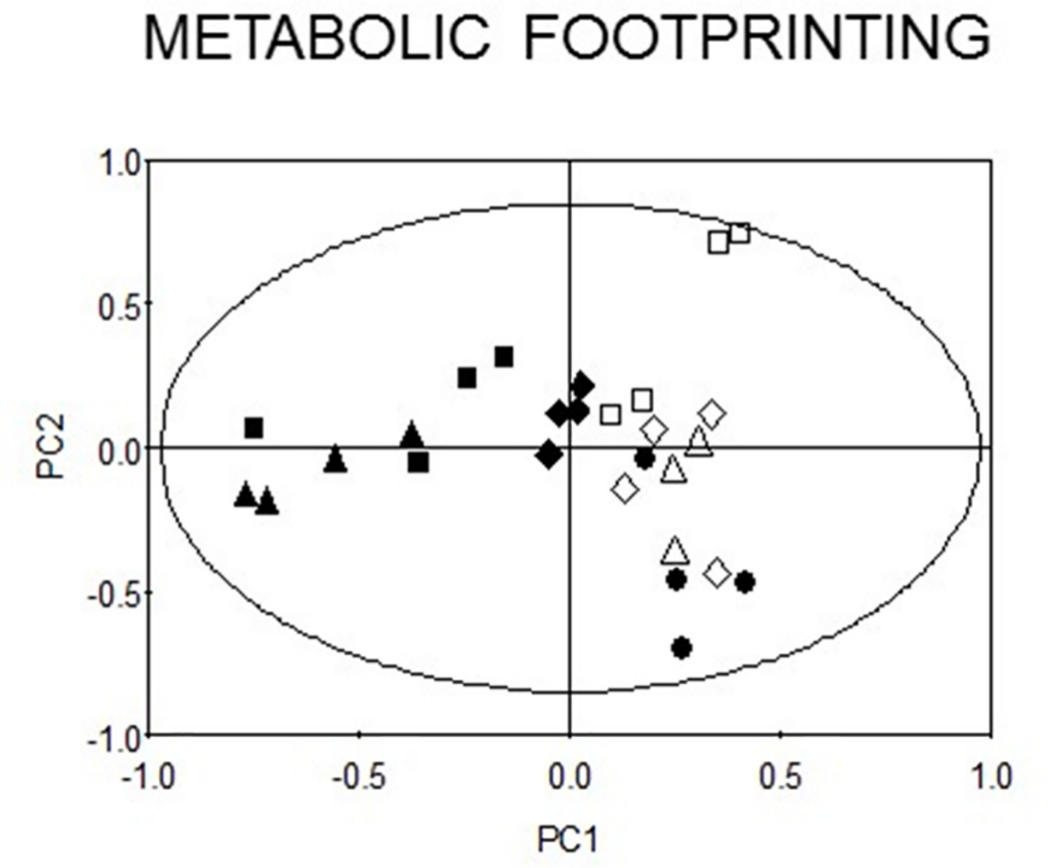
^1^H-NMR metabolomic analysis of QS mutants and AZM-treated wild-type *P*. *aeruginosa*. The figure shows a PCA scores plot of the ^1^H-NMR spectroscopy data after processing to remove resonance contributions deriving from AZM. The cumulative R^2^ value for PC1 and PC2 is 44%. **Symbols**: *lasI* mutant ■, *rhlI* mutant ▲, *pqsR* mutant ◆, untreated PAO1 ●, PAO1 treated with 2 μg/ml AZM △, PAO1 treated with 8 μg/ml AZM ◊, PAO1 treated with 32 μg/ml AZM □.

Recent work suggests that QS has a large impact on the metabolome [51]. Therefore, we also examined whether AZM might affect the metabolic footprint of *P*. *aeruginosa*. Stationary-phase culture supernatants of PAO1 ± AZM and the *lasI*/*rhlI*/*pqsR* mutants were analysed using ^1^H-NMR spectroscopy. The spectra were divided into bins and analysed using PCA (Fig 5). Bins containing spectral signatures from AZM were excluded from the analysis, as were the corresponding regions in the spectra of the various QS mutants and untreated wild-type samples. Principal component 1 (PC1), accounting for 25% of the total variation in the dataset, primarily separated the *lasI* and the *rhlI* mutants from the wild-type, with intermediate separation observed for the *pqsR* mutant. This confirms that QS does indeed have a significant impact on the metabolic footprint, as recently reported [51]. In contrast, the AZM-treated samples primarily separated along PC2 (accounting for 19% of the total variation in the dataset), with little or no trend along PC1. This indicates that while AZM treatment does affect the exometabolome, these effects seem to be largely independent of its effect(s) on QS.

### Identification of AZM modulated proteins

We identified a selection of the proteins that were modulated by AZM treatment using MS/MS. Although we endeavoured to pick as many modulated spots as possible, not all of these came back as “clean” hits due to e.g., spot overlap. Since the identity of the specific protein that is modulated in such “mixed spots” cannot be ascertained with certainty, they were not analysed further. The identities of those “clean hit” protein spots that were modulated by AZM alone in the cell-associated and extracellular fractions are shown in S1 and S2 Tables, respectively. Note that these Tables include spots identified as modulated based on both BVA and PCA analyses (as indicated), whereas the spot numbers in Fig 3 only reflect those spots that were significantly modulated (p≤0.01) in the BVA. The positions and identities of all of the AZM-modulated spots are mapped onto the 2D gels shown in Fig 2.

We also identified a selection of protein spots that were modulated by AZM treatment and in one or more of the QS mutants (S3 Table). Of particular note is the fact that AZM down-regulated the extracellular protein, PA4625 (CdrA). CdrA plays a key role in maintaining biofilm architecture [52], and its down-regulation is consistent with our previous report showing that sub-MIC AZM suppresses biofilm formation in AGSY growth media [35]. To further investigate this, we used the mini-CTX*lacZ* system [44] to generate a stable chromosomal insertion in PAO1 in which the expression of a promoter-less *lacZ* ORF was placed under the control of the *cdrA* promoter. The β-galactosidase activity was measured in the presence or absence of 32 μg/ml AZM in AGSY media. Two hours after the culture had entered the stationary phase (i.e., at a similar time point as the samples for proteomic analysis were harvested) the β-galactosidase activity in the AZM-treated samples was decreased 4-fold (*data not shown*).

Considering the rather sparse sampling that necessarily accompanies this type of 2D-DiGE-based proteomic analysis, there was a reassuring overlap in the identities of the spots modulated by AZM here and those reported by earlier workers. Indeed, of the 20 AZM-modulated spots that we identified in the secretome, 10 of these (AotJ, CysP, DsbA, PA0572, CbpD, FliC, FliD, LasB and GroEL) were also identified by Nalca *et al* as being AZM-modulated in their growth conditions [20]. However, the overlap between the cell-associated proteome spots identified in both studies was much smaller. Indeed, just 1 out of the 21 cell-associated AZM-modulated proteins that we identified was also identified by Nalca *et al* (a spot corresponding to the Fpr protein).

### Identification of QS-modulated proteins in the cell-associated fraction

Given that QS had a large effect on the exometabolome of PAO1 (Fig 5) and that these metabolic perturbations are likely to be brought about by QS-dependent physiological changes inside the cell, we felt that it would be instructive to also identify a selection of the QS-modulated spots from the cell-associated fraction (S4 Table). The intracellular QS-regulated proteome has received far less attention than the QS-regulated secretome, yet we now know that QS has a profound impact on the metabolic status of the cell [51]. We identified 41 proteins (some of which were represented by multiple spots). Only one of these (the taurine binding protein, TauA) was identified as QS-regulated in a previous global proteomic analysis of cell-associated proteins [39], although several others (HsiC3, CbpD, PhzS, RmlA) have been implicated as QS-controlled in more focussed studies [22,23,38,53]. Consistent with the role of QS in redirecting metabolism, many of the QS-modulated proteins were predicted to be involved in metabolic processes. However, perhaps one of the more intriguing QS-modulated cell-associated proteins that we identified was MagG; a newly-characterized α2-macroglobulin-like protein with likely protease inhibitory activity. MagD has been previously reported to be regulated by RetS and RsmA [54]. It is also worth noting that Fpr is represented on the “QS but not AZM-modulated” list (S4 Table) and on the “AZM but not QS-modulated list” (S1 Table). This is because Fpr was represented by more than one spot. One of these spots was modulated by QS but not AZM and the other was modulated by AZM but not QS. This suggests that QS and AZM may influence post-translational processing of Fpr.

### Azithromycin decreases secreted protease activity in *Serratia marcescens*

**Fig 6:**
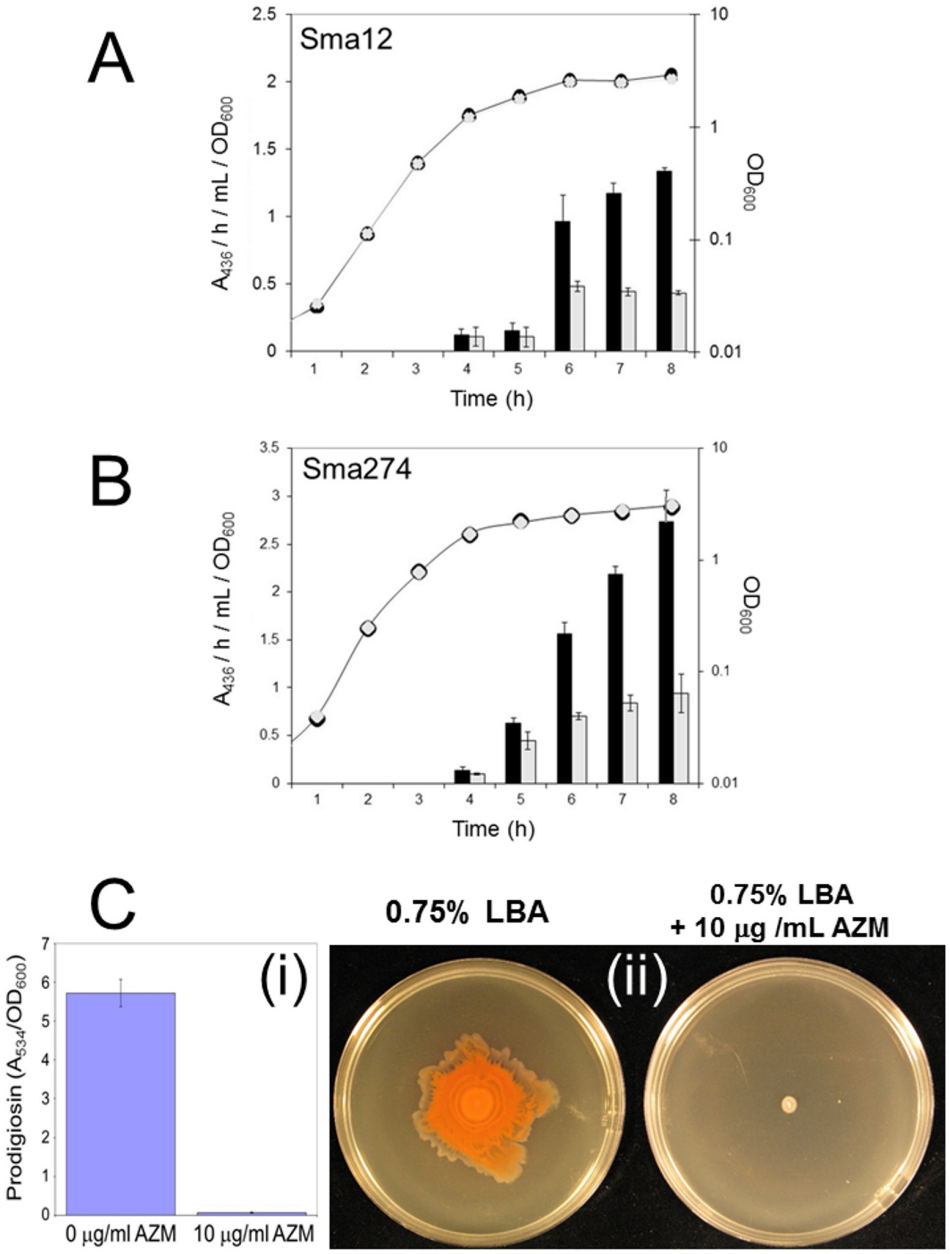
Impact of sub-MIC AZM on virulence phenotypes of *Serratia marcescens*. Secreted protease was measured through the growth curve of the QS^+^ *Serratia marcescens* strain Sma12 (panel A) and the QS^-^ strain Sma274 (panel B) in the absence (black bars/symbols) or presence (grey bars/symbols) of 10 μg/ml AZM. Panel C; impact of 10 μg/ml AZM on (i) prodigiosin production in planktonic cultures of Sma274, and (ii) surface swarming by Sma274.

Our data show that AZM affects only a small QS sub-regulon in *P*. *aeruginosa*, and that the antibiotic cannot be considered a generic QS inhibitor (at least, in our growth conditions). This made us wonder whether the previously-reported impact of AZM on QS might be a “red herring” and that AZM actually interferes with virulence *via* mechanisms that are mainly independent of its effect(s) on QS. To test this possibility further, we examined whether AZM affected virulence factor production in another Gram-negative species that is well-known for secreting exoenzymes; *Serratia marcescens* (Sma). Like *P*. *aeruginosa*, this pathogen produces copious quantities of secreted protease, and in many strains this protease production is QS-regulated [46]. Indeed, and consistent with the situation in *P*. *aeruginosa*, when we tested whether AZM could suppress secreted protease production in a QS^+^ strain (Sma12) of *S*. *marcescens*, it did (Fig 6, upper panel). However, the advantage of working with *S*. *marcescens* is that unlike the situation in *P*. *aeruginosa*, where exoprotease production is strictly QS-regulated, some strains of *S*. *marcescens* (such as Sma274) lack a functional QS system but still secrete protease. Intriguingly, previous work has shown that when the QS system of Sma12 is introduced into Sma274 (by phage-mediated generalized transduction), the production of secreted protease by Sma274 comes under the control of the newly-acquired QS machinery [46]. Consequently, protease production in Sma274 is *potentially* QS regulatable, even though it is not normally *de facto* regulated in this way. At concentrations <32 μg/ml, AZM had little or no effect on the growth of Sma274. Nevertheless, we found that 10 μg/ml AZM suppressed secreted protease production by Sma274 (Fig 6, middle panel). AZM (10 μg/ml) also completely abolished production of the secondary metabolite, prodigiosin, and blocked swarming in Sma274 (Fig 6, lower panel). Assuming that AZM does not act *via* completely different mechanisms in *P*. *aeruginosa* and *S*. *marcescens* we conclude that AZM can suppress a very similar range of phenotypes in these different organisms (including secondary metabolite production, motility and secreted protease) and that this suppression may not be dependent on the presence of a functional QS system.

## Discussion

Despite the fact that our results, and those of others [18–21] show inhibition of QS-dependent phenotypes by AZM, a detailed side-by-side analysis of protein expression profiles reveals that sub-MIC AZM modulates only a small fraction of the QS-dependent proteome. AZM affected a roughly similar proportion of QS-regulated proteins in both the intracellular and secretome fractions. Intriguingly, in the secretome, AZM had a greater influence over rhl-dependent protein expression than it did over the las-controlled proteome, even though the las-dependent secretome consists of approximately twice as many protein spots (S1 Fig). Given that many of the known *P*. *aeruginosa* virulence factors are under the control of the rhl system [55,56], disruption of this sub-component of the QS system (or of a sub-regulon associated with it) would be expected to have a disproportionately larger effect on virulence than disruption of the las system. In this regard, we also note that Gödeke *et al* have shown that AZM-mediated “drop off” can have a particularly large effect on the translation of *rhlR* transcripts (and presumably, therefore also the RhlR-controlled regulon) [37].

Two previous microarray analyses have investigated AZM action in *P*. *aeruginosa*. One of these [57] focussed on the effect of AZM on biofilms, although given the large differences between the transcriptome and/or secretome of biofilms and planktonic cultures [47,58,59], it seems unlikely that any meaningful comparisons can be made between the output of that study and the current one. In the second study, based on a comparison of the effects of sub-MIC AZM on *P*. *aeruginosa* grown in BHI medium with previously-published lists of QS-regulated transcripts, Nalca *et al* reported that AZM exhibited extensive QS antagonistic activity in planktonic *P*. *aeruginosa* [20]. However, and as outlined earlier, the same workers also acknowledged the potential limitations of their interpretation; limitations that are confirmed by our data showing that AZM actually affects only a small proportion of the total QS-regulated proteome. This notwithstanding, it is worth emphasizing that (i) there was a good overlap in the identified AZM-modulated extracellular proteins identified by Nalca *et al* and the current study, and (ii) while AZM affected only a small fraction of the global QS regulon, over half of the AZM-modulated spots were under the control of QS. Moreover, some of the jointly-modulated spots that were affected by both AZM and QS in the current work—such as CdrA, LasB and PA0572 (an uncharacterized M60-like protease that appears with regularity among lists of QS-regulated extracellular proteins)—are likely to have an impact on the virulence of the organism.

The current study, like earlier ones investigating the impact of AZM *in vitro*, is not without limitations, and these need to be acknowledged. For example, the growth medium (alanine-glycerol-salts) used in this study may not mimic the environment in the CF lung. However, we note that L-alanine, which is the principle carbon source in AGS medium, is rapidly consumed by *P*. *aeruginosa* [51], and is known to be preferentially catabolized by *P*. *aeruginosa* above other carbon sources (including carbohydrates) [60]. Moreover, a *dadA* mutant, which is unable to catabolize L-alanine *in vitro*, displays impaired competitiveness *in vivo* in a rat lung infection model, indicating that L-Ala is also an important nutrient in the rat lung environment [60]. Another possible limitation of the current work is that *P*. *aeruginosa* is thought to grow as biofilm-like aggregates in the CF airways [61] whereas we analysed the effect(s) of AZM in planktonic cultures. Given that biofilms and planktonic cultures are known to display distinctly different transcriptomic and proteomic profiles, this too is a variable that may confound direct comparison with the action of AZM in CF airways [47,58]. Interestingly, biofilms of PAO1 have been reported to produce more BHL than OdDHL, whereas the opposite is the case with planktonic cultures [62]. A similar, BHL-dominated profile, is also seen in CF sputum (presumably reflecting the biofilm-like growth of *P*. *aeruginosa* in sputum [62]). Given that our data show that AZM has a large effect on a small rhl-dependent sub-regulon, this *may* go some way towards explaining the therapeutic impact of sub-MIC AZM *in vivo*. Another possibility that needs considering is that AZM might primarily act on the host rather than the bacteria. For example, it is possible that one or more host-derived factors may modulate the QS system *in vivo*, thereby enhancing its sensitivity to AZM-dependent inhibition. Alternatively, we note that in our *in vitro* analyses there were no phagocytic cells such as leucocytes, yet it is well established that due to its dibasic nature, AZM is rapidly taken up and concentrated by these immune cells and thence delivered directly to the site of infection [63]. Clearly, the unusual pharmacodynamics of the AZM/leucocyte partnership might also contribute towards the apparent sub-MIC effects of this drug *in vivo*.

This is not the first report to question whether sub-MIC AZM affects QS, although to our knowledge, it is the first to examine the effects of AZM side-by-side with defined QS mutants. Phelan *et al* recently used MALDI imaging mass spectrometry to examine the impact of AZM on secondary metabolite production by several strains of *P*. *aeruginosa* (PAO1, PA14 and a clinical isolate, FLR01) [64]. After adjusting for cell growth, those workers reported that sub-MIC AZM had no significant impact on a range of QS-regulated secondary metabolites, including pyoverdine, alkyl quinolones, pyocyanin and rhamnolipids. Indeed, they reported that many of these compounds actually increased in abundance in AZM-stressed cells.

In addition to suppressing secreted protease production by *P*. *aeruginosa*, AZM also suppressed secreted protease production by *Serratia marcescens*, and this suppression was independent of the presence of a functional QS system. Assuming that AZM acts in a mechanistically similar way in both species, these data suggest that the antibiotic does not target virulence factor production by blocking QS *per se*. Interestingly, recent work has shown that AZM also improves lung function in CF patients who are not carrying *P*. *aeruginosa* (but who may carry other bacterial species), suggesting that inhibition of secreted virulence factor production by *P*. *aeruginosa* may not necessarily be the only means by which AZM mediates its therapeutic effects [65,66]; the virulence phenotypes of other species may also be affected. It is also worth noting that the QS regulon of *P*. *aeruginosa* itself varies substantially from strain to strain, and that the “core” QS regulon is actually rather small [67].

Where possible, we identified the proteins that were modulated in response to AZM. In the cell-associated proteome, the largest category of modulated proteins was “central and intermediary metabolism”. This suggests that AZM is likely to perturb metabolism and is consistent with the observed effects of AZM on the ^1^H-NMR spectroscopy-visible metabolic footprint of *P*. *aeruginosa*. Similarly, in both the cell-associated and extracellular proteome fractions, proteins involved in the uptake of small molecules were also disproportionately represented in the AZM-modulated dataset. Again, these changes could impact on the composition of the metabolome. Another group of proteins found to be modulated in response to AZM treatment were stress-related, including SodM, DsbA, GroEL, PA1789 (a member of the UspA-family of stress-endurance proteins), RplY (ribosomal protein L25, with homology to the CTC stress-induced protein) and the probable antioxidant protein LsfA. We note that Nalca *et al* also reported AZM-dependent modulations in DsbA, GroEL, and the superoxide dismutase SodB [20]. Taken together, these results suggest that sub-MIC concentrations of AZM induce a stress response in *P*. *aeruginosa*.

AZM modulated proteins are involved in the motility of *P*. *aeruginosa*. The expression of FliC and FliD (involved in flagellar-mediated motility) were decreased by AZM. Consistent with this, flagella-driven swimming motility has been previously shown to be suppressed by sub-MIC AZM [20]. However, not all secreted AZM-modulated proteins showed such neat correlations in our dataset. Twitching motility has been shown to be suppressed by sub-MIC AZM [68], yet the pilus protein PilY1 displayed increased expression in the presence of AZM. [In this regard, Nalca *et al* also noted increased expression of *pilA* and *pilH* transcripts in the presence of AZM [20].] Moreover, the *Pseudomonas aeruginosa* small protease (PasP) and the probable haemagglutinin, PA0041 also both showed increased expression in the presence of sub-MIC AZM. Clearly, the effects of sub-MIC AZM on virulence factor production are complex, and potentially affect both QS-regulated (S3 Table) and non QS-regulated (S2 Table) secreted virulence factors.

## Conclusions

We have examined the proteomic changes brought about by a range of clinically-relevant concentrations of AZM, and compared these to the changes brought about by defined QS mutations grown alongside in the same conditions. Our results show that AZM perturbs QS but does not globally influence it, and this is further supported by metabolic footprint analyses. We conclude that the other proposed mechanistic activities of AZM (e.g., promoting “drop off”, inhibiting *gacA* expression, or the simple remodelling of microbial communities through the killing-off of sensitive organisms) likely play a more central role in the therapeutic activity of sub-MIC AZM than inhibition of QS *per se*. In particular, we note that the disproportionate effect of sub-MIC AZM on the rhl component of the QS system is consistent with the AZM-induced *rhlR* transcript “drop off” hypothesis proposed by Gödeke *et al* [37].

## Supporting Information

S1 Fig Overlap(s) between the QS and AZM-modulated proteome. The figure shows Venn diagrams indicating inter-relationships between the protein spots that were significantly (p≤0.01) modulated in the individual QS mutants (*lasI*, *rhlI* and *pqsR*) or AZM-treated wild-type (inclusive of all concentrations of AZM tested) compared with untreated PA01.
(TIF)

S1 Table QS-independent effects of AZM on the intracellular proteome. Single hit proteins modulated when PAO1 is treated with AZM (2, 8 or 32 μg/ml) but not (p≤0.01) in the different QS mutants (*lasI*, *rhlI* and *pqsR*) compared with untreated PAO1. *Listed proteins were identified either by DeCyder Biological Variation Analysis (BVA, p≤0.01) or multivariate analysis (Principle Components Analysis, PCA, see Materials and Methods) and ontologically classified according to the Pseudomonas Genome Project database (www.pseudomonas.com/). The directional trend and magnitude of the observed change in spot intensity is shown. For the spots identified as significantly modulated in the BVA (univariate) analysis, the directional trend is shown only for those spots modulated with a p value ≤0.01. For the protein spots identified through multivariate (PCA) analysis, note that the p value is >0.01. Where no change in spot intensity was observed, this is indicated with a “-”symbol. The *P*. *aeruginosa* number refers to an annotated open reading frame in the PAO1 genome sequence (21). MASCOT ions score is -10*log(P), where P is the probability that the observed match is a random event. Individual ions scores >52 indicate identity or extensive homology (p≤0.05). The number of peptides matched, the percent coverage for each polypeptide, the predicted pI and nominal mass are also shown for each protein.
(DOCX)

S2 Table QS-independent effects of AZM on the extracellular proteome. Single hit proteins modulated when PAO1 is treated with AZM (2, 8 or 32 μg/ml) but not (p≤0.01) in the different QS mutants (*lasI*, *rhlI* and *pqsR*) compared to untreated PAO1. See legend to S1 Table for details.
(DOCX)

S3 Table Intracellular and extracellular proteins modulated in the QS mutants (*lasI*, *rhlI* and *pqsR*) and in PAO1 following AZM treatment. Single hit proteins modulated (BVA, p≤0.01) in one or more of the QS mutants (*lasI*, *rhlI* and *pqsR*) and when PAO1 is treated with AZM (2, 8 or 32 μg/ml) compared with untreated PAO1.
(DOCX)

S4 Table Intracellular proteins modulated in the QS mutants (*lasI*, *rhlI* and *pqsR*). Single hit proteins modulated (BVA, p≤0.01) in one or more of the QS mutants (*lasI*, *rhlI* and *pqsR*) compared with untreated PAO1.
(DOCX)
