# Supplementary figures and images for: Impact of Azithromycin on the Quorum Sensing-Controlled Proteome of Pseudomonas aeruginosa
Source: PLoS One. 2016 Jan 25;11(1):e0147698. doi: 10.1371/journal.pone.0147698 (PMC4726577; doi:10.1371/journal.pone.0147698)

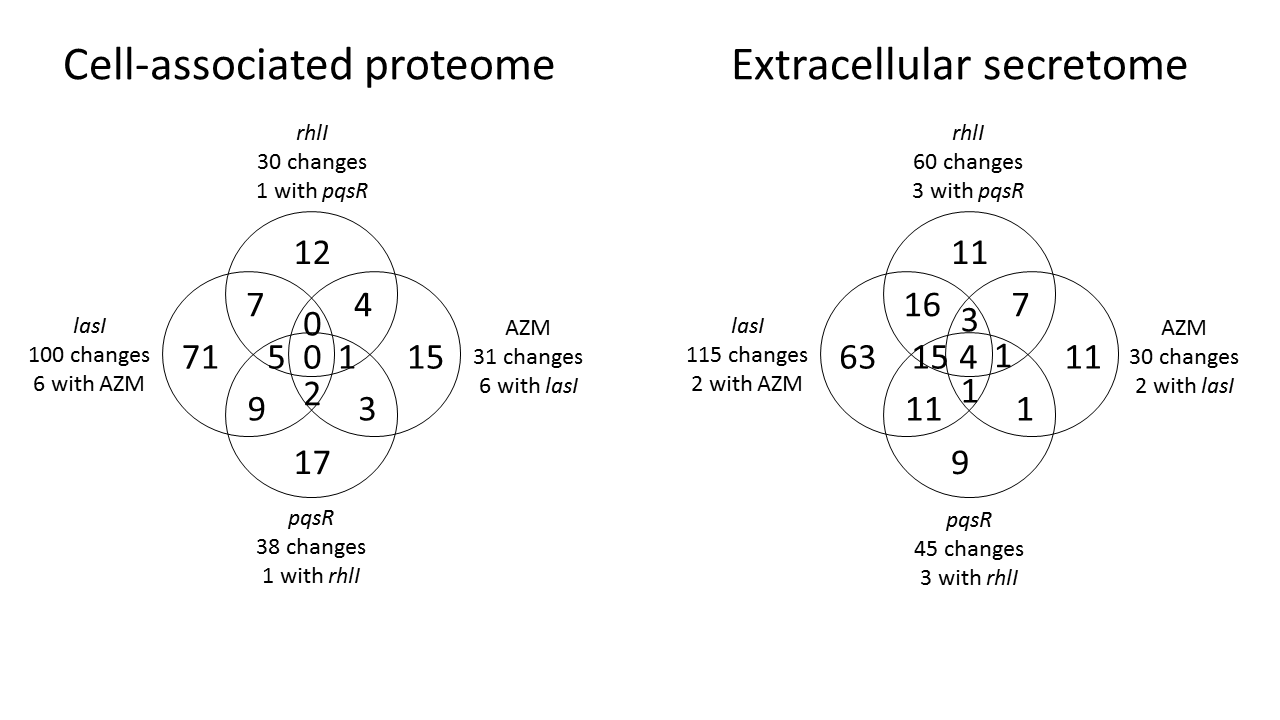

Supplement: S1 Fig — The figure shows Venn diagrams indicating inter-relationships between the protein spots that were significantly (p≤0.01) modulated in the individual QS mutants (lasI, rhlI and pqsR) or AZM-treated wild-type (inclusive of all concentrations of AZM tested) compared with untreated PA01. (TIF) [file pone.0147698.s001.tif]
