# Supplementary material for: Impact of Azithromycin on the Quorum Sensing-Controlled Proteome of Pseudomonas aeruginosa
Source: PLoS One. 2016 Jan 25;11(1):e0147698. doi: 10.1371/journal.pone.0147698 (PMC4726577; doi:10.1371/journal.pone.0147698)
Supplement: S1 Table — Single hit proteins modulated when PAO1 is treated with AZM (2, 8 or 32 μg/ml) but not (p≤0.01) in the different QS mutants (lasI, rhlI and pqsR) compared with untreated PAO1. *Listed proteins were identified either by DeCyder Biological Variation Analysis (BVA, p≤0.01) or multivariate analysis (Principle Components Analysis, PCA, see Materials and Methods) and ontologically classified according to the Pseudomonas Genome Project database (www.pseudomonas.com/). The directional trend and magnitude of the observed change in spot intensity is shown. For the spots identified as significantly modulated in the BVA (univariate) analysis, the directional trend is shown only for those spots modulated with a p value ≤0.01. For the protein spots identified through multivariate (PCA) analysis, note that the p value is >0.01. Where no change in spot intensity was observed, this is indicated with a “-”symbol. The P. aeruginosa number refers to an annotated open reading frame in the PAO1 genome sequence (21). MASCOT ions score is -10*log(P), where P is the probability that the observed match is a random event. Individual ions scores >52 indicate identity or extensive homology (p≤0.05). The number of peptides matched, the percent coverage for each polypeptide, the predicted pI and nominal mass are also shown for each protein. (DOCX) [file pone.0147698.s002.docx]

| **S1 Table : Cell-associated proteins that are modulated by AZM and not QS.** | | | | | | | | | | | | |
| --- | --- | --- | --- | --- | --- | --- | --- | --- | --- | --- | --- | --- |
| Protein | PA number | Gene name | Modulation in the presence of: | | | MASCOT  ions score | Sequence  coverage (%) | No. of  peptides | pI | Nominal mass (Da) | BVA | PCA |
|  |  |  | (2 μg/ml AZM) | (8 μg/ml AZM) | (32 μg/ml AZM) |  |  |  |  |  |  |  |
| **Central Intermediary Metabolism** | | | | | | | | | | | | |
| Acetyl-CoA synthetase | PA0887 | *acsA* | - | - | ↓ 1.48 | 547 | 11 | 7 | 5.91 | 72230 | * |  |
| Aspartokinase | PA0904 | *lysC* | - | - | ↓ 2.14 | 252 | 34 | 10 | 4.88 | 23377 | * |  |
| Probable NADH-ubiquinone oxidoreductase | PA1883 | *yhfP* | - | - | ↓ 1.47 | 682 | 48 | 46 | 5.39 | 34701 | * |  |
| Ferredoxin-NADP reductase | PA3397 | *fpr* | ↓ 1.10 | ↓ 1.50 | ↓ 1.88 | 234 | 34 | 10 | 5.65 | 29556 |  | * |
| Probable dehydrogenase | PA4079 | N/A | - | ↓ 1.38 | ↓ 1.42 | 329 | 44 | 14 | 5.27 | 24418 | * |  |
| Probable fumarase | PA4333 | *fumA* | ↓ 1.08 | ↑ 1.25 | ↑ 1.57 | 249 | 10 | 7 | 5.89 | 49674 |  | * |
| **Transport of Small Molecules** | | | | | | | | | | | | |
| Arginine/ornithine binding protein | PA0888 | *aotJ* | ↓ 1.13 | ↓ 1.38 | ↓ 1.63 | 236 | 46 | 10 | 5.29 | 24207 |  | * |
| Sulfate binding protein of ABC transporter | PA1493 | *cysP* | - | ↓ 1.44 | ↓ 1.52 | 520 | 57 | 20 | 7.77 | 36495 | * |  |
| Fe(III)-pyochelin outer membrane receptor | PA4221 | *fptA* | ↑ 1.43 | ↓ 1.24 | ↓ 1.95 | 952 | 33 | 26 | 5.86 | 80000 |  | * |
| Outer membrane receptor for iron transport | PA4515 | *piuA* | - | - | ↓ 1.87 | 734 | 22 | 19 | 5.72 | 82401 |  | * |
| **Adaptation, Protection, Chaperones and Heat Shock** | | | | | | | | | | | | |
| UspA-like hypothetical protein | PA1789 | N/A | - | ↓ 1.61 | ↓ 2.53 | 710 | 61 | 28 | 5.36 | 31482 | * |  |
| Probable antioxidant protein | PA3450 | *lsfA* | ↑ 1.10 | ↓ 1.36 | ↓ 1.42 | 134 | 26 | 5 | 5.29 | 24207 |  | * |
| **Uncharacterized/hypothetical** | | | | | | | | | | | | |
| Hypothetical protein | PA2464 | N/A | ↓ 1.20 | ↓ 1.56 | ↓ 1.99 | 117 | 21 | 3 | 4.86 | 17615 |  | * |
| Hypothetical protein | PA3785 | N/A | - | ↓ 1.65 | ↓ 1.69 | 271 | 35 | 10 | 6.28 | 16990 |  | * |
| **Translation, Post-translational Modification, Degradation** | | | | | | | | | | | | |
| Ribosomal Protein L25 | PA4671 | *rplY* | - | ↑ 1.28 | - | 220 | 28 | 15 | 5.81 | 22006 |  | * |
