# Supplementary material for: Impact of Azithromycin on the Quorum Sensing-Controlled Proteome of Pseudomonas aeruginosa
Source: PLoS One. 2016 Jan 25;11(1):e0147698. doi: 10.1371/journal.pone.0147698 (PMC4726577; doi:10.1371/journal.pone.0147698)
Supplement: S2 Table — Single hit proteins modulated when PAO1 is treated with AZM (2, 8 or 32 μg/ml) but not (p≤0.01) in the different QS mutants (lasI, rhlI and pqsR) compared to untreated PAO1. See legend to S1 Table for details. (DOCX) [file pone.0147698.s003.docx]

| **S2 Table : Secreted proteins that are modulated by AZM and not QS.** | | | | | | | | | | | | |
| --- | --- | --- | --- | --- | --- | --- | --- | --- | --- | --- | --- | --- |
| Protein | PA number | Gene name | Modulation in the presence of: | | | MASCOT  ions score | Sequence  coverage (%) | No. of  peptides | pI | Nominal mass (Da) | BVA | PCA |
|  |  |  | (2 μg/ml AZM) | (8 μg/ml AZM) | (32 μg/ml AZM) |  |  |  |  |  |  |  |
| **Uncharacterized/hypothetical** | | | | | | | | | | | | |
| Hypothetical protein | PA1579 | N/A | - | ↑ 1.98 | ↑ 2.41 | 341 | 31 | 7 | 7.71 | 22211 |  | * |
| Hypothetical protein | PA3445 | N/A | - | ↑ 1.82 | ↑ 4.33 | 304 | 26 | 7 | 8.55 | 35163 |  | * |
| **Adaptation, Protection, Chaperones and Heat Shock** | | | | | | | | | | | | |
| GroEL (spot 1) | PA4385 | *groEL* | ↓ 1.34 | ↑ 6.46 | ↑ 4.05 | 700 | 33 | 15 | 5.04 | 57093 |  | * |
| GroEL (spot 2) | PA4385 | *groEL* | ↑ 1.24 | ↑ 8.74 | ↑ 7.32 | 867 | 38 | 22 | 5.04 | 57093 |  | * |
| Thiol-disulfide interchange protein precursor | PA5489 | *dsbA* | - | ↑ 3.51 | ↑ 3.92 | 134 | 18 | 3 | 5.98 | 23403 |  | * |
| **Transport of Small Molecules** | | | | | | | | | | | | |
| Sulfate binding protein precursor (spot 1) | PA0283 | *sbp* | - | - | ↑ 2.76 | 85 | 7 | 2 | 8.45 | 36411 | * |  |
| Sulfate binding protein precursor (spot 2) | PA0283 | *sbp* | - | - | ↑ 2.52 | 300 | 29 | 8 | 8.45 | 36411 | * |  |
| Heme-transport protein | PA4708 | *phuT* | - | - | ↑ 3.97 | 558 | 48 | 10 | 6.86 | 31045 | * |  |
| Sulfate binding protein of ABC transporter | PA1493 | *cysP* | - | ↑ 3.23 | ↑ 3.96 | 343 | 32 | 9 | 7.77 | 36495 | * |  |
| Arginine/ornithine binding protein | PA0888 | *aotJ* | - | - | ↑ 3.65 | 385 | 34 | 11 | 6.43 | 28192 | * |  |
| Polyamine binding protein | PA0300 | *spuD* | - | ↑4.76 | - | 435 | 26 | 9 | 6.97 | 40718 | * |  |
| Probable binding protein of ABC transporter | PA1342 | N/A | - | - | ↑ 3.15 | 372 | 29 | 13 | 8.30 | 33147 | * |  |
| Outer membrane porin | PA2760 | *oprQ* | ↑ 1.15 | ↑ 1.41 | ↑ 2.03 | 316 | 16 | 6 | 5.54 | 46865 |  | * |
| **Secreted factors, motility and attachment** | | | | | | | | | | | | |
| Probable hemagglutinin | PA0041 | N/A | ↓ 1.28 | ↑ 3.57 | ↑ 2.80 | 134 | 1 | 2 | 5.30 | 361891 |  | * |
| Secreted protease | PA0423 | *pasP* | ↓ 1.34 | ↑ 2.95 | ↑ 3.78 | 486 | 45 | 45 | 6.09 | 20764 |  | * |
| Flagellin type B | PA1092 | *fliC* | - | ↓ 2.05 | - | 1105 | 43 | 53 | 5.40 | 49213 | * |  |
| Flagellar capping protein | PA1094 | *fliD* | ↓ 1.25 | ↓ 1.97 | ↓ 1.46 | 54 | 2 | 1 | 6.52 | 49420 |  | * |
| Type 4 fimbrial biogenesis protein | PA4554 | *pilY1* | ↓ 1.12 | ↑ 1.24 | ↑ 2.20 | 126 | 2 | 3 | 7.62 | 127676 |  | * |
