# Supplementary material for: Impact of Azithromycin on the Quorum Sensing-Controlled Proteome of Pseudomonas aeruginosa
Source: PLoS One. 2016 Jan 25;11(1):e0147698. doi: 10.1371/journal.pone.0147698 (PMC4726577; doi:10.1371/journal.pone.0147698)
Supplement: S3 Table — Single hit proteins modulated (BVA, p≤0.01) in one or more of the QS mutants (lasI, rhlI and pqsR) and when PAO1 is treated with AZM (2, 8 or 32 μg/ml) compared with untreated PAO1. (DOCX) [file pone.0147698.s004.docx]

| **S3 Table : Proteins that are modulated (p≤0.01) by AZM and QS.** | | | | | | | | | | | |
| --- | --- | --- | --- | --- | --- | --- | --- | --- | --- | --- | --- |
| Protein | PA number | Gene name | Modulation in the presence of: | | | Modulation in QS mutant | MASCOT  ions score | Sequence  coverage (%) | No. of  peptides | pI | Nominal mass (Da) |
|  |  |  | (2 μg/ml AZM) | (8 μg/ml AZM) | (32 μg/ml AZM) |  |  |  |  |  |  |
| ***Cell-associated proteome*** | | | | | | | | | | | |
| **Adaptation, protection, chaperone and heat shock proteins** | | | | | | | | | | | |
| Probable glutathione S-transferase | PA1033 | *yfcG* | - | ↓ 1.54 | ↓ 2.07 | ↑ 1.30 in *lasI* mutant, ↓ 2.4 in *pqsR* mutant | 157 | 13 | 8 | 6.30 | 24512 |
| Trigger factor (spot 1) | PA1800 | *tig* | - | ↑ 1.85 | - | ↑ 2.30 in *lasI* mutant | 1057 | 65 | 34 | 4.83 | 48552 |
| Trigger factor (spot 2) | PA1800 | *tig* | - | ↑ 1.69 | - | ↑ 2.03 in *lasI* mutant | 1421 | 76 | 67 | 4.83 | 48552 |
| Superoxide dismutase | PA4468 | *sodM* | - | ↑ 2.54 | ↑ 4.19 | ↑ 2.04 in *lasI* mutant | 215 | 31 | 6 | 5.81 | 22492 |
| **Transcription** | | | | | | | | | | | |
| N utilization substance protein A | PA4745 | *nusA* | - | ↑ 1.37 | - | ↑ 1.58 in *lasI* mutant | 894 | 39 | 46 | 4.55 | 54682 |
| ***Extracellular proteome*** | | | | | | | | | | | |
| **Uncharacterized/hypothetical** | | | | | | | | | | | |
| Hypothetical protein | PA0572 | N/A | - | ↓ 2.41 | - | ↓ 23.86 in *lasI* mutant | 2416 | 55 | 361 | 6.12 | 100607 |
| **Secreted Factors (toxins, enzymes, alginate)** | | | | | | | | | | | |
| Cyclic di-GMP regulated TPS partner A | PA4625 | *cdrA* | - | - | ↓ 2.11 | ↓ 1.97 in *lasI* mutant, ↓ 1.92 in *pqsR* mutant, ↓ 3.49 in *rhlI* mutant | 3223 | 29 | 367 | 4.94 | 219739 |
| Chitin binding protein precursor | PA0852 | *cbpD* | - | ↑ 3.15 | - | ↓ 38.89 in *lasI* mutant, ↓ 14.35 in *pqsR* mutant, ↓ 15.17 in *rhlI* mutant | 273 | 17 | 5 | 6.65 | 42375 |
| Elastase (spot 1) | PA3724 | *lasB* | - | ↓ 2.85 | - | ↓ 141.46 in *lasI* mutant, ↓ 8.04 in *pqsR* mutant, ↓ 9.01 in *rhlI* mutant | 308 | 26 | 12 | 6.28 | 53882 |
| Elastase (spot 2) | PA3724 | *lasB* | - | ↓ 2.44 | - | ↓ 238.97 in *lasI* mutant, ↓ 5.86 in *pqsR* mutant, ↓ 6.23 in *rhlI* mutant | 405 | 47 | 17 | 6.28 | 53882 |
