# Supplementary material for: Impact of Azithromycin on the Quorum Sensing-Controlled Proteome of Pseudomonas aeruginosa
Source: PLoS One. 2016 Jan 25;11(1):e0147698. doi: 10.1371/journal.pone.0147698 (PMC4726577; doi:10.1371/journal.pone.0147698)
Supplement: S4 Table — Single hit proteins modulated (BVA, p≤0.01) in one or more of the QS mutants (lasI, rhlI and pqsR) compared with untreated PAO1. (DOCX) [file pone.0147698.s005.docx]

| **S4 Table : Cell-associated proteins that are modulated (p≤0.01) by QS.** | | | | | | | | | | |
| --- | --- | --- | --- | --- | --- | --- | --- | --- | --- | --- |
| Protein | PA number | Gene name | Modulation in: | | | MASCOT  ions score | Sequence  coverage (%) | No. of  peptides | pI | Nominal mass (Da) |
|  |  |  | *lasI* mutant | *pqsR* mutant | *rhlI* mutant |  |  |  |  |  |
| Hypothetical protein (probable fumaryloacetoacetase) | PA0318 | *hpcE* | ↑ 2.22 | - | - | 221 | 47 | 11 | 5.36 | 23943 |
| Hypothetical protein (probable phosphatase) | PA0469 | N/A | ↑ 2.21 | - | - | 643 | 52 | 24 | 4.94 | 32345 |
| Hypothetical protein (probable nitronate monoxygenase) | PA0660 | N/A | ↑ 3.68 | - | ↑ 1.85 | 378 | 27 | 10 | 6.24 | 34800 |
| Hypothetical protein | PA0754 | N/A | ↑ 3.13 | - | - | 517 | 36 | 16 | 5.58 | 35010 |
| Chitin binding protein precursor (4 spots) | PA0852 | *cbpD* | ↓ 2.97,  ↓ 4.53,  ↓ 6.17,  ↓ 12.06 | ↓ 2.86,  ↓ 3.99,  ↓ 4.21,  ↓ 5.48 | ↓ 2.68,  ↓ 2.57,  ↓ 4.02,  ↓ 5.17 | 103,  639,  428,  532 | 12,  64,  47,  53 | 3,  32,  21,  23 | 6.65 | 42375 |
| N-succinylglutamate 5-semialdehyde dehydrogenase | PA0895 | *aruC* | ↑ 1.80 | - | - | 1140 | 68 | 49 | 5.63 | 43834 |
| Outer membrane porin precursor (4 spots) | PA0958 | *oprD* | ↓ 2.44  -  ↓ 2.38  ↓ 3.65 | ↓ 4.06  ↓ 4.14  ↓ 3.66  ↓ 4.69 | -  -  -  - | 352,  416,  511,  655 | 24,  28,  28,  34 | 12,  11,  16,  25 | 4.96 | 48331 |
| Hypothetical protein (2 spots) | PA1011 | N/A | ↑ 5.98,  ↑ 3.84 | -  - | -  - | 467,  756 | 34,  59 | 10,  23 | 4.92 | 43087 |
| Trigger factor | PA1800 | *tig* | ↑ 1.79 | - | - | 944 | 62 | 34 | 4.83 | 48552 |
| H3 T6S locus protein | PA2366 | *hsiC3* | ↓ 2.07 | - | - | 72 | 7 | 3 | 5.56 | 55179 |
| Pyoverdine synthase F (2 spots) | PA2396 | *pvdF* | ↑ 5.26,  ↑ 5.06 | - | - | 149,  314 | 22,  27 | 8,  16 | 5.15 | 30993 |
| Ferripyoverdine receptor | PA2398 | *fpvA* | ↑ 2.25 | - | - | 1320 | 49 | 64 | 5.08 | 87745 |
| Probable class III aminotransferase | PA2413 | *pvdH* | ↑ 5.10 | ↑ 2.78 | - | 896 | 54 | 54 | 6.03 | 50412 |
| Hypothetical protein | PA2575 | N/A | ↑ 2.18 | - | - | 357 | 50 | 17 | 5.96 | 22208 |
| Cysteine synthase A | PA2709 | *cysK* | ↑ 1.58 | - | - | 661 | 64 | 27 | 6.24 | 34402 |
| Electron transfer flavoprotein α subunit | PA2951 | *etfA* | ↑ 1.77 | - | - | 677 | 77 | 66 | 4.98 | 31404 |
| 3-phosphoshikimate-1-carboxyvinyltransferase prephenate | PA3164 | N/A | ↑ 1.31 | - | - | 641 | 26 | 17 | 5.8 | 79670 |
| Phosphogluconate dehydratase | PA3191 | *edd* | ↓ 1.49 | - | - | 508 | 31 | 18 | 6.24 | 65597 |
| Hypothetical protein | PA3302 | N/A | - | - | ↑ 2.51 | 367 | 58 | 14 | 5.24 | 16891 |
| Cytochrome P450 | PA3331 | N/A | - | - | ↑ 2.20 | 468 | 34 | 20 | 5.54 | 46483 |
| Ferredoxin-NADP^+^ reductase | PA3397 | *fpr* | ↓ 1.88 | - | - | 663 | 65 | 42 | 5.65 | 29556 |
| Transcriptional regulator | PA3587 | *metR* | - | ↑ 1.69 | - | 451 | 45 | 16 | 5.55 | 34480 |
| Elongation factor TS (2 spots) | PA3655 | *tsf* | ↑ 1.73  ↑ 1.98 | -  - | -  - | 498,  712 | 43,  58 | 17,  53 | 5.22 | 30691 |
| Peptide chain release factor | PA3903 | *prfC* | ↓ 1.89 | - | - | 489 | 21 | 15 | 5.60 | 60227 |
| Hypothetical protein (probable lipoprotein); 2 spots | PA3931 | N/A | -  - | ↓ 2.11  ↓ 2.13 | -  - | 433,  507 | 48,  48 | 14,  19 | 7.74 | 28084 |
| Probable periplasmic taurine binding protein | PA3938 | *tauA* | - | - | ↑ 3.14 | 302 | 29 | 8 | 6.99 | 35838 |
| Probable thioredoxin with tetratricopeptide (TTP)-like domain | PA4061 | *ybbN* | ↑ 1.63 | - | - | 462 | 43 | 11 | 4.63 | 32086 |
| FAD-dependent monooxygenase | PA4217 | *phzS* | - | ↓ 1.89 | - | 579 | 33 | 22 | 5.69 | 43732 |
| GroEL | PA4385 | *groEL* | ↑ 1.28 | - | - | 1279 | 61 | 75 | 5.04 | 57093 |
| α2-macroglobulin-like protein | PA4489 | *magD* | ↓ 1.98 | - | - | 895 | 19 | 26 | 5.48 | 167326 |
| Peptidyl-prolyl *cis*-*trans* isomerase | PA4572 | *fklB* | ↑ 2.51 | - | - | 173 | 34 | 5 | 4.78 | 21782 |
| Ferric iron-binding periplasmic protein | PA4687 | *hitA* | ↑ 2.02 | - | - | 422 | 35 | 14 | 5.54 | 36184 |
| Heme/hemoglobin uptake outer membrane receptor (2 spots) | PA4710 | *phuR* | ↑ 1.57  ↑ 3.27 | ↑ 1.28  - | -  ↑ 1.78 | 900,  1061 | 36,  44 | 27,  56 | 5.72 | 84370 |
| DnaK (5 spots) | PA4761 | *dnaK* | ↑ 1.72  ↑ 1.77  ↑ 1.63  ↑ 1.69  ↑ 1.62 | -  -  -  -  - | -  -  -  -  - | 788,  804,  1371,  1382,  1395 | 36,  36,  50,  44,  45 | 27,  22,  47,  49,  52 | 4.79 | 68475 |
| Hypothetical extracellular solute binding protein | PA5137 | N/A | - | ↓ 1.36 | - | 254 | 39 | 8 | 6.63 | 27974 |
| Glucose 1-phosphate thymidylyltransferase | PA5163 | *rmlA* | - | ↓ 1.55 | - | 237 | 24 | 8 | 5.22 | 32494 |
| Arginine deiminase (3 spots) | PA5171 | *arcA* | -  -  - | -  -  - | ↑ 5.29  ↑ 5.33  ↑ 4.14 | 510,  927,  1015 | 29,  58,  57 | 15,  40,  82 | 5.52 | 46820 |
| Ornithine carbamoyltransferase (2 spots) | PA5172 | *arcB* | -  - | -  - | ↑ 4.53  ↑ 3.44 | 319,  588 | 34,  48 | 13,  23 | 6.14 | 38124 |
| Probable transcarboxylase | PA5435 | *oadA* | ↓ 1.42 | - | - | 823 | 45 | 27 | 5.59 | 66282 |
| Class II (cobalamin-dependent) ribonucleotide-diphosphate reductase | PA5497 | *nrdJa* | ↓ 1.73 | - | - | 241 | 14 | 9 | 5.76 | 83260 |
| Probable extracellular zinc-binding protein | PA5498 | *znuA* | - | ↓ 1.54 | - | 218 | 24 | 9 | 5.78 | 33758 |
